# Cause‐specific mortality rates in patients with diabetes according to comorbid macro‐ and microvascular complications: BioBank Japan Cohort

**DOI:** 10.1002/edm2.181

**Published:** 2020-08-27

**Authors:** Hiroshi Yokomichi, Akiko Nagai, Makoto Hirata, Mie Mochizuki, Reiji Kojima, Zentaro Yamagata, BioBank Japan Project

**Affiliations:** ^1^ Department of Health Sciences University of Yamanashi Chuo Japan; ^2^ Department of Public Policy Institute of Medical Science The University of Tokyo Tokyo Japan; ^3^ Genetic Medicine and Services National Cancer Center Hospital Tokyo Japan; ^4^ Department of Pediatrics University of Yamanashi Chuo Japan

**Keywords:** cancer, cause of death, diabetic vascular complications, mortality rate

## Abstract

**Objective:**

This study aimed to compare cause‐specific mortality rates in patients with type 2 diabetes with and without various vascular complications.

**Methods:**

In Japanese hospitals, we followed up 30 834 patients with a mean age of 64.4 (standard deviation [SD]: 11.1) years. Patients were followed up from 2003 to 2007 for a median of 7.5 (interquartile range: 6.1‐9.7) years. We calculated cause‐specific mortality rates (number of deaths/1000 person‐years) and confounder‐adjusted hazard ratios in patients with macrovascular disease and in those with diabetic nephropathy, neuropathy and retinopathy, allowing for overlap of complications.

**Results:**

All‐cause mortality rate was highest (51.4) in the nephropathy group, followed by the macrovascular disease group (45.2), the neuropathy group (39.5), the retinopathy group (38.7) and the nonvascular complication group (18.1). In the nephropathy group, morality rates of ischaemic heart, cerebrovascular, and infectious diseases and cancer were also highest among the groups. However, the cancer mortality rate was similar among the vascular complication groups. Relative to the nonvascular complication group, covariate‐adjusted hazard ratios for ischaemic heart and cerebrovascular disease mortality were triple to quadruple in the macro‐ and microvascular complication groups. All‐cause mortality rates rose exponentially according to age.

**Conclusion:**

Highest risks of all‐cause, cancer, and ischaemic heart, infectious, and cerebrovascular disease mortality were determined in Japanese patients with diabetic nephropathy. Although cancer is the primary cause of death in Japanese patients with diabetes, cancer mortality rates are similar among those with and without vascular complications.

## Graphical abstract

Patients with diabetic nephropathy have the highest mortality rates of all causes, cancer, and macrovascular and infectious diseases. The primary cause of death of patients with diabetes with and without all vascular complications was cancer. The cancer mortality rate was similar, regardless of whether vascular complications were present.

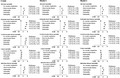

## 1 INTRODUCTION

The number of patients with diabetes has been rapidly increasing worldwide. The International Diabetes Federation estimated that 4.2 million people aged between 20 and 79 years died from diabetes in 2019, and diabetes accounted for 10.7% of global all‐cause mortality of people of this age.^1^ The mortality rate of patients with diabetes is extremely high. Internationally, all‐cause mortality rates are 29 and 23 per 1000 person‐years in men and women with diabetes (mean age of 58 years), respectively, while they are 12 and 7 per 1000 person‐years, respectively, in the general population (mean age of 55 years).^2^

In Western countries and China, the primary cause of death in patients with type 2 diabetes is vascular diseases, followed by cancer.^2^, ^3^ In contrast, Taiwanese and Japanese patients with type 2 diabetes primarily die from cancer followed by vascular diseases.^4^, ^5^ Therefore, the ranking of causes of death in patients varies depending on the country. Since treatment of diabetes and self‐management of patients improve with time, mortality rates of nonmacrovascular diseases will increase in patients with diabetes. However, evidence of cause‐specific mortality rates in patients in Asian countries is insufficient.

The prevalence of vascular complications in patients with diabetes also varies, depending on the country and its data sources. Having diabetic vascular complications indicates progression of diabetes and thus may predict life expectancy of patients with diabetes. Indeed, some studies have shown that patients with diabetes with macrovascular complications have double to triple the risk of all‐cause mortality.^6^, ^7^ Having microvascular complications of diabetes is also considered to shorten the life expectancy of patients directly and by indirectly restricting activities of daily living. However, cause‐specific mortality rates among Asian patients with microvascular complications are not well known.

In this study, we examined a hospital‐based cohort that included patients with known prevalent diabetes to determine all‐cause and cause‐specific mortality rates of patients with diabetic complications. We also calculated the hazard ratios to compare the mortality risks among those with diabetic complications.

## 2 MATERIAL AND METHODS

### 2.1 Participants

Between the fiscal years 2003 and 2007, the Tailor‐Made Medical Treatment Program with the BioBank Japan Project registered patients with type 2 diabetes at 66 hospitals. The study profile has been published elsewhere.^8^ In brief, we followed participants with one of 47 targeted diseases in these hospitals from June 2003 to March 2013. We observed survival of the patients and new onset of diseases. During the observation period, when participants without diabetes developed diabetes, we enrolled and followed them up as patients with diabetes. Medical coordinators in the participating hospitals collected information on survival or death of the patients using medical records. If the patients had not visited the hospitals for longer than 1 year, the medical coordinators requested residence registrations to municipalities to certify their life or death. The causes of deaths were investigated by requesting vital statistics by the Vital Statistics Act. Consequently, the cohort achieved a 97% follow‐up rate.

### 2.2 Vascular complication categories

We collected information on diabetic complications using a standardized questionnaire from the medical records at enrolment.^8^ We identified vascular complications as follows. (a) The macrovascular disease complication group included patients with a history of acute myocardial infarction, stable or unstable angina pectoris, heart failure, cerebral infarction, cerebral haemorrhage, subarachnoid haemorrhage, cerebral arterial aneurysm, aortic aneurysm and peripheral artery disease. (b) The diabetic nephropathy group included patients with complicated diabetic nephropathy of stage 1 (prenephropathy), stage 2 (incipient nephropathy), stage 3 (overt nephropathy), stage 4 (kidney failure) and stage 5 (any status of continued dialysis therapy).^9^ In this hospital setting, where only a subset of patients was followed up by diabetologists, most of the patients in this category had stage 2 or 3 nephropathy. (c) The diabetic neuropathy group included patients with diabetic polyneuropathy. (d) The diabetic retinopathy group included patients with simple, preproliferative and proliferative diabetic retinopathy.^10^ This study permitted overlapping of vascular complications, which is usual in patients with diabetes. We aimed to calculate mortality risk according to each complication rather than the combination. If patients had two or more vascular complications, they were included in all of the relevant vascular complication groups.

### 2.3 Measurements

We collected clinical information of the patients at baseline by interviewing the patients and reviewing medical records.^8^ The information included a past history of diseases, medications, use of tobacco and alcohol, and the habit of physical activity. Serum glycated haemoglobin A1c (HbA1c) levels, which were measured in the scale of The Japanese Diabetes Society between 2003 and 2007, were converted to those in the National Glycohemoglobin Standardization Program (NGSP, %) and the International Federation of Clinical Chemistry and Laboratory Medicine (IFCC, mmol/mol).^11^ The duration of diabetes was calculated as that from the date of onset or diagnosis of diabetes to the time of enrolment. Causes of death were identified according to the 10th revision of the International Statistical Classification of Diseases and Related Health Problems (ICD‐10).^12^ Causes of death were grouped into ischaemic heart disease (I20‐25), cerebrovascular disease (I60‐69), aortic dissection (I70‐79), cancer (C00‐97, D00‐48), infectious disease (A00‐B99, E14.4, J00‐42, J80‐86, G00‐09, L00‐05, L08, N30‐77, R02), renal failure (N00‐19, E14.2) and gastrointestinal disease (K00‐93). Death from gangrene (A41.9, E14.4, L00‐05, L08, R02) was a subset of death from infectious disease.

### 2.4 Statistical analysis

We described proportions of male sex, diabetic medication, cancer or macrovascular comorbidity, diabetes therapy and smoking, drinking, and physical activity habits, means of age, body mass index (BMI), systolic blood pressure, serum total cholesterol levels, HbA1c, disease and follow‐up duration at baseline in each vascular complication group. The rates of all‐cause, ischaemic heart, cerebrovascular, infectious and gastrointestinal diseases, aortic dissection, cancer, gangrene and renal failure mortality were calculated as the number of deaths divided by 1000 person‐years. We also calculated the all‐cause mortality rates of the vascular complication groups from the ages of 45 to 79 years in both sexes. We calculated Kaplan‐Meier estimates of all‐cause mortality. The log‐rank test was used to assess the significance of differences between the vascular and nonvascular complication groups. To compare cause‐specific mortality rates among the vascular complication groups relative to the nonvascular complication group, we calculated crude and adjusted hazard ratios (HRs). Model 1 had adjusted HRs for sex, age and duration of diabetes. Model 2 had adjusted HRs for sex, age, duration of diabetes, comorbid cancer, medications of diabetes, hypertension, and dyslipidemia, habits of smoking and alcohol drinking, and frequency of physical activity. We also evaluated the statistical significance of HRs between two groups in a pairwise manner. Statistical analyses were performed using SAS statistical software (version 9.4, SAS Institute, Cary, NC, USA). R statistical software (version 2.15.3, R Project for Statistical Computing, Vienna, Austria) was used to generate Kaplan‐Meier estimates. All reported p values were two‐sided, and *P* < .05 indicates a significant difference.

## 3 RESULTS

### 3.1 Patients’ characteristics

Table 1 shows the number of vascular complications, age, HbA1c levels, disease duration and follow‐up duration among the vascular complication groups in 30 834 patients. Among the groups, the proportions of male sex ranged from 60.8% to 69.2%, mean age from 61.8 to 67.8 years, mean BMI from 23.8 to 24.5 kg/m^2^ and mean HbA1c levels from 7.4% to 7.7% (57.1‐60.3 mmol/mol). The median follow‐up period was 7.8 years (interquartile range [IQR]: 5.2‐9.5 years) in the macrovascular disease group, 7.6 years (IQR: 4.8‐9.4 years) in the nephropathy group, 8.0 years (IQR: 5.7‐9.3 years) in the neuropathy group, 8.0 years (5.7‐9.7 years) in the retinopathy group and 8.3 years (6.7‐9.8 years) in the nonvascular complication group. In the macrovascular disease group, 3007 (23.3%), 3042 (23.6%) and 2519 (19.5%) patients also had diabetic nephropathy, neuropathy and retinopathy, respectively. In the diabetic nephropathy group, 3007 (48.7%), 2639 (42.7%) and 2131 (34.5%) patients had macrovascular disease, neuropathy and retinopathy, respectively. In the diabetic neuropathy group, 3042 (45.5%), 2639 (39.5%) and 2341 (35.0%) patients had macrovascular disease, nephropathy and retinopathy, respectively. In the diabetic retinopathy group, 2519 (43.3%), 2131 (36.6%) and 2341 (40.2%) patients had macrovascular disease, nephropathy and neuropathy, respectively.

**TABLE 1:** Baseline characteristics of patients with type 2 diabetes with macro‐ and microvascular complications

Characteristics	All patients	Macrovascular disease	Nephropathy	Neuropathy	Retinopathy	No macro‐ or microvascular complications
Number	30 834	12 904 (41.8)	6174 (20.0)	6689 (21.7)	5823 (18.9)	10 850 (35.2)
Overlapping vascular complications, n (%)						
Macrovascular		–	3007 (48.7)	3042 (45.5)	2519 (43.3)	
Nephropathy		3007 (23.3)	–	2639 (39.5)	2131 (36.6)	
Neuropathy		3042 (23.6)	2639 (42.7)	–	2341 (40.2)	
Retinopathy		2519 (19.5)	2131 (34.5)	2341 (35.0)	–	
Age, years	64.4 (11.1)	67.8 (9.7)	64.9 (11.4)	64.7 (10.3)	64.7 (10.8)	61.8 (11.7)
Number of men (%)	19 830 (64.3)	8927 (69.2)	4114 (66.6)	4121 (61.6)	3715 (63.8)	6593 (60.8)
Body mass index, kg/m^2^	24.3 (4.0)	24.2 (3.7)	24.4 (4.2)	23.8 (3.8)	24.0 (3.9)	24.5 (4.1)
Missing data	2234 (7.2)	1017 (7.9)	525 (8.5)	439 (6.6)	470 (8.1)	698 (6.4)
SBP, mmHg	134 (17)	135 (18)	138 (19)	136 (18)	135 (18)	132 (16)
Missing data	4732 (15.3)	2056 (15.9)	965 (15.6)	954 (14.3)	978 (16.8)	1612 (14.9)
Total cholesterol, mg/dL	206 (43)	198 (40)	205 (47)	204 (46)	207 (44)	214 (40)
Missing data	21 459 (69.6)	8225 (63.7)	4182 (67.7)	4879 (72.9)	4236 (72.7)	7904 (72.8)
HbA1c, %	7.4 (1.4)	7.4 (1.4)	7.5 (1.5)	7.7 (1.5)	7.6 (1.6)	7.4 (1.3)
HbA1c, mmol/mol	57.7 (15.4)	57.1 (15.1)	57.9 (16.7)	60.3 (16.6)	59.9 (17.5)	57.1 (14.6)
Missing data	9031 (29.3)	3099 (24.0)	1948 (31.6)	1993 (29.8)	1683 (28.9)	3553 (32.7)
Diabetes therapy						
Sulfonylurea	11 274 (36.6)	4672 (36.2)	2024 (32.8)	2383 (35.6)	2086 (35.8)	3945 (36.4)
Alpha‐glucosidase inhibitor	7491 (24.3)	3158 (24.5)	1476 (23.9)	1660 (24.8)	1466 (25.2)	2518 (23.2)
Insulin	5021 (16.3)	2007 (15.6)	1568 (25.4)	2046 (30.6)	1610 (27.7)	1221 (11.3)
Metformin	3337 (10.8)	1129 (8.8)	669 (10.8)	880 (13.2)	695 (11.9)	1233 (11.4)
Thiazolidinedione	2488 (8.1)	881 (6.8)	454 (7.4)	442 (6.6)	458 (7.9)	976 (9.0)
Glinide	1280 (4.2)	447 (3.5)	241 (3.9)	222 (3.3)	259 (4.5)	519 (4.8)
Number of drugs	11067 (35.9)	5004 (38.8)	2063 (33.4)	1916 (28.6)	1630 (28.0)	4094 (37.7)
Duration of diabetes, years	8.4 (8.3)	9.6 (9.0)	11.3 (9.4)	12.1 (9.5)	11.0 (9.2)	6.0 (6.5)
Smoking status						
Current smoker	7692 (25.0)	2697 (20.9)	1521 (24.6)	1575 (23.6)	1564 (26.9)	3036 (28.0)
Former smoker	9272 (30.1)	4971 (38.5)	1952 (31.6)	1969 (29.4)	1706 (29.3)	2586 (23.8)
Never smoker	13 870 (45.0)	5236 (40.6)	2701 (43.8)	3145 (47.0)	2553 (43.8)	5228 (48.2)
Currently drinking alcohol	11 587 (38.8)	4511 (36.2)	2049 (34.2)	2233 (34.6)	2073 (36.5)	4415 (42.0)
Physical activity						
3 times/wk or more	2160 (7.5)	791 (6.6)	328 (5.7)	374 (6.0)	350 (6.4)	905 (9.0)
1‐2 times/wk	2246 (7.8)	759 (6.3)	329 (5.7)	367 (5.9)	338 (6.2)	989 (9.8)
No habit of exercise	24278 (84.6)	10 433 (87.1)	5124 (88.6)	5477 (88.1)	4796 (87.5)	8190 (81.2)
Missing data	2150 (7.0)	921 (7.1)	393 (6.4)	471 (7.0)	339 (5.8)	766 (7.1)
Cancer or macrovascular comorbidity	14 693 (47.7)	12 904 (100)	3295 (53.4)	3375 (50.5)	2735 (47.0)	1165 (10.7)
Follow‐up duration, years	7.6 (2.8)	7.2 (3.0)	7.0 (3.1)	7.4 (2.9)	7.4 (2.9)	7.8 (2.6)

Note

Data are shown as mean (standard deviation) unless indicated as number (%).

### 3.2 Cause‐specific and age‐specific mortality

Table 2 shows the mortality rates of major causes in the vascular complication groups. When we compared groups with and without vascular complications, patients with nephropathy had the highest point estimates of mortality rates from all causes, cancer, infectious disease, renal failure, ischaemic heart disease, cerebrovascular disease, gastrointestinal disease, gangrene and aortic dissection. The most frequent cause of death in the nephropathy group was cancer, followed by infectious disease and renal failure, ischaemic heart disease, cerebrovascular disease and gastrointestinal disease. The most frequent cause of death in the macrovascular disease and retinopathy groups was cancer, followed by infectious disease, ischaemic heart disease, cerebrovascular disease and renal failure. The most frequent cause of death in the neuropathy group was cancer, followed by infectious disease, ischaemic heart disease, renal failure, cerebrovascular disease and gastrointestinal disease. In contrast, the most frequent cause of death in the nonvascular complication group was cancer, followed by infectious disease, gastrointestinal disease, ischaemic heart disease, cerebrovascular disease and renal failure. Table 3 shows sex‐ and age‐specific all‐cause mortality rates of patient age between 45 and 79 years. In both sexes, the all‐cause mortality rates rose in an exponential manner. In both sexes at any age, the all‐cause mortality rate was highest in the nephropathy group, among all groups. In all of the sex and age profiles, the point estimate of the all‐cause mortality rate was higher in male patients than in female patients.

**TABLE 2:** Cause‐specific and age‐specific mortality rates in type 2 diabetic patients with and without vascular complications

Mortality rate, number of deaths/1000 person‐years	Macrovascular disease	Nephropathy	Neuropathy	Retinopathy	No vascular complications	All
All patients						
All‐cause mortality rate	45.2	51.4	39.5	38.7	18.1	31.2
Ischaemic heart disease mortality rate	5.6	6.2	4.5	3.9	0.9	3.1
Cerebrovascular disease mortality rate	3.8	3.8	3.0	3.0	0.8	2.2
Aortic dissection mortality rate	0.6	0.7	0.3	0.4	0.1	0.3
Cancer mortality rate	8.5	8.6	8.3	7.5	7.2	7.9
Infectious disease mortality rate	5.8	6.4	4.6	4.6	1.8	3.6
Gangrene mortality rate in infectious disease	1.0	1.4	0.9	0.8	0.2	0.6
Renal failure mortality rate	2.8	6.4	3.4	2.9	0.2	1.6
Gastrointestinal disease mortality rate	1.7	2.4	1.5	1.9	1.1	1.4

**TABLE 3:** Sex‐ and age‐specific all‐cause mortality rates in type 2 diabetic patients with and without vascular complications

	No of event/no of patients						Mortality rate, no. of death/1000 person‐years					
	Macrovascular disease	Nephropathy	Neuropathy	Retinopathy	No vascular complication	All	Macrovascular disease	Nephropathy	Neuropathy	Retinopathy	No vascular complication	All
Men												
At 45‐49 y of age	16/133	10/75	14/107	18/173	20/407	78/895	15.1	16.4	16.2	0.2	6.2	11.0
At 50‐54 y of age	35/313	11/137	37/227	74/337	52/718	209/1732	13.4	10.0	20.3	29.1	9.1	15.2
At 55‐59 y of age	87/615	58/243	83/393	120/572	102/1078	450/2901	18.0	32.1	27.8	27.8	12.1	20.1
At 60‐64 y of age	162/855	75/247	123/453	179/643	151/1111	690/3309	23.8	44.1	36.3	37.4	17.3	27.1
At 65‐70 y of age	256/1035	110/318	174/527	219/685	193/1060	952/3625	32.7	49.2	45.0	44.3	23.3	35.0
At 70‐74 y of age	365/1038	131/302	194/479	233/568	230/872	1153/3259	49.3	67.6	60.5	59.3	35.6	50.3
At 75‐79 y of age	279/636	115/196	124/253	172/360	162/466	852/1911	65.6	105.8	78.2	76.0	51.7	69.1
Women												
At 45‐49 y of age	2/22	2/26	4/59	5/55	6/179	19/341	12.7	9.6	7.9	10.9	4.0	6.7
At 50‐54 y of age	5/68	3/58	10/111	6/142	16/365	40/744	8.8	6.3	10.8	5.0	5.2	6.4
At 55‐59 y of age	13/166	12/97	19/194	23/228	44/654	111/1339	9.5	16.0	11.2	12.1	8.1	10.0
At 60‐64 y of age	32/268	21/113	33/238	44/317	47/672	177/1608	14.7	23.1	17.1	16.7	8.4	13.4
At 65‐70 y of age	61/357	24/114	62/238	77/371	64/759	288/1928	21.4	27.0	24.0	26.1	10.4	18.7
At 70‐74 y of age	98/448	47/134	80/314	104/391	93/610	422/1897	28.2	51.2	33.2	34.9	20.0	29.3
At 75‐79 y of age	118/407	50/126	90/246	97/294	96/453	451/1526	39.7	62.0	51.6	46.5	28.9	41.3

### 3.3 Crude survival curves and adjusted hazard ratios

**FIGURE 1:**
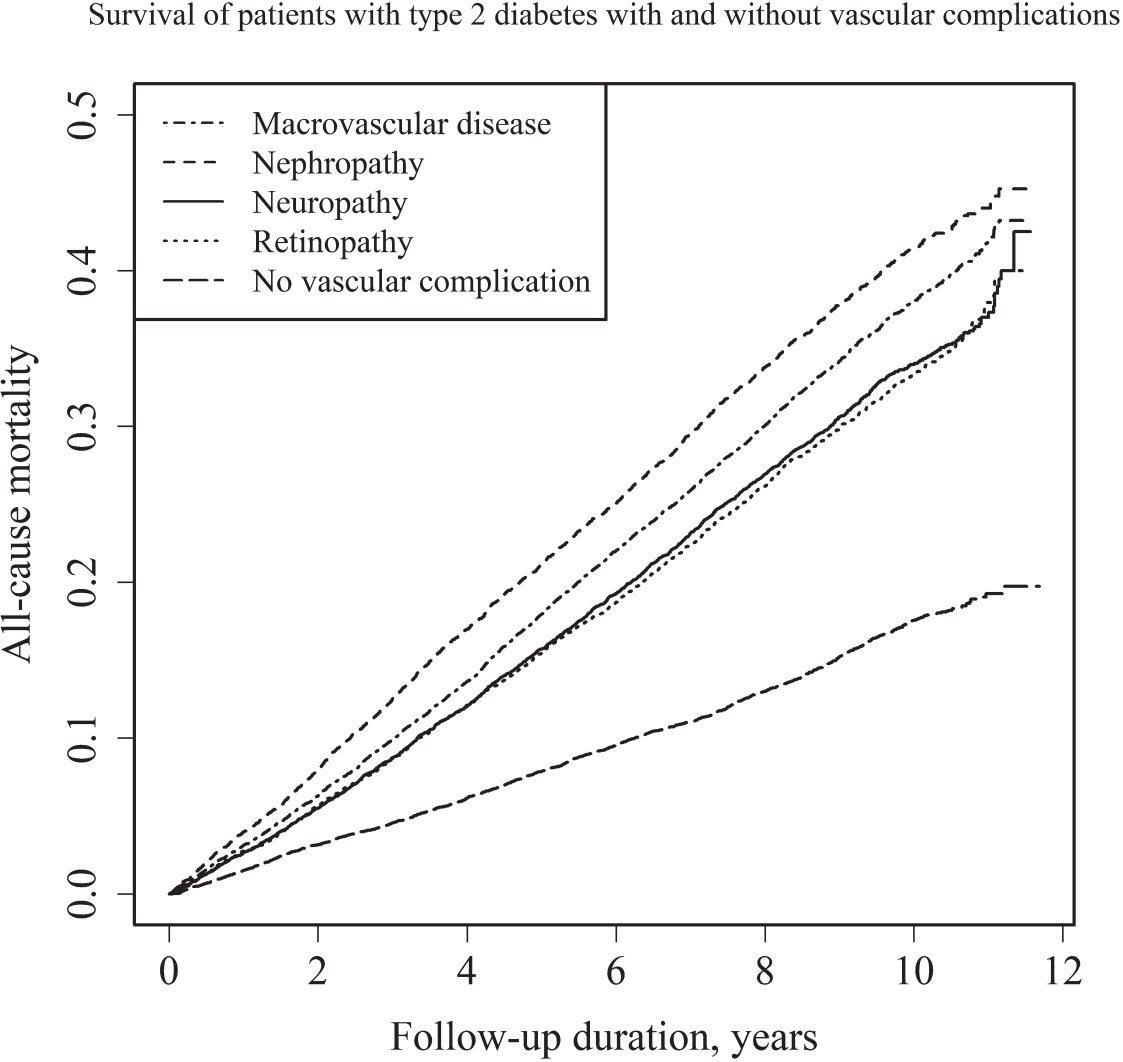
Mortality of Japanese patients with diabetes and vascular complications

**FIGURE 2:**
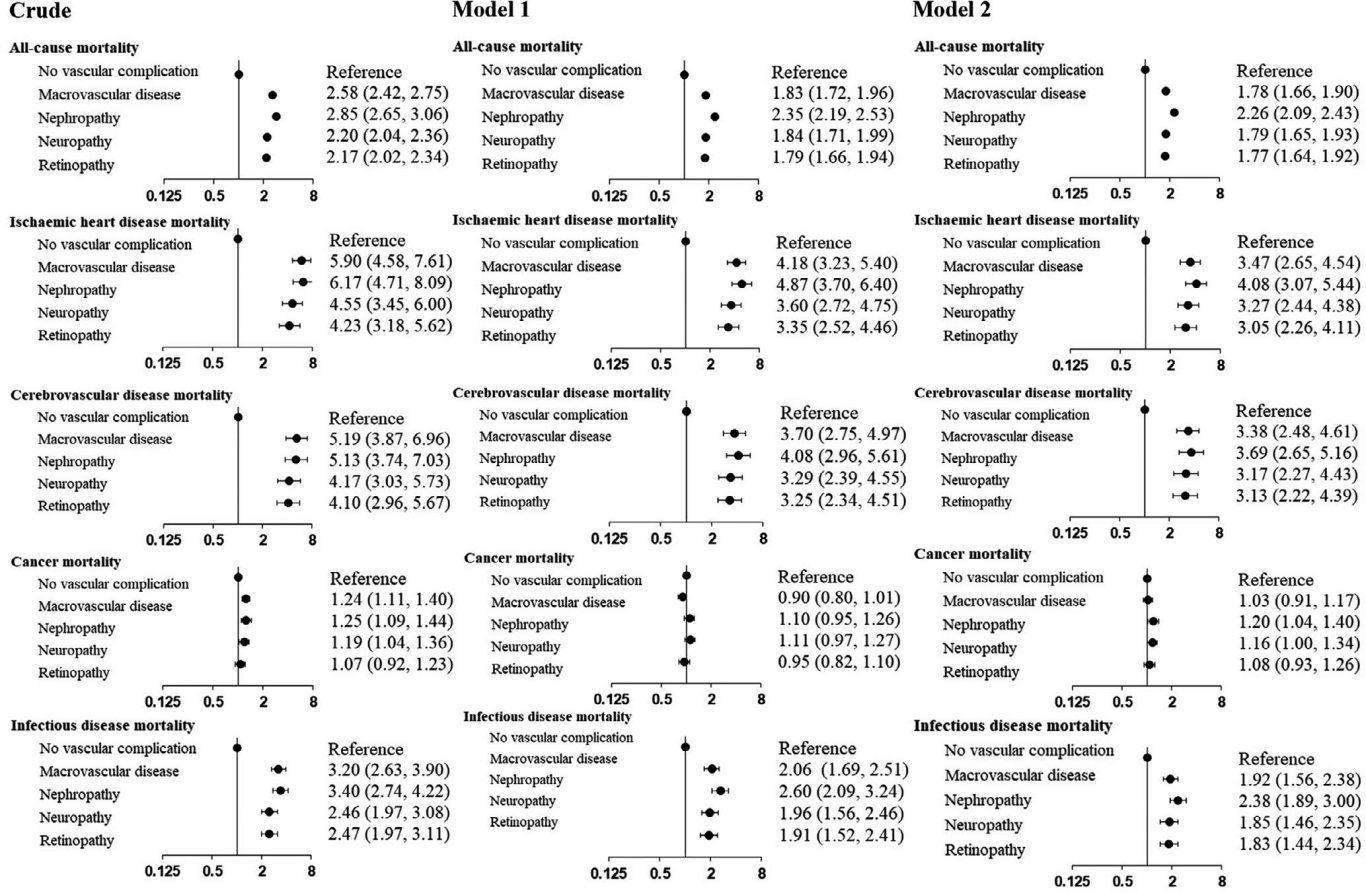
Hazard ratios (95% confidence intervals) of cause‐specific mortality in Japanese patients with type 2 diabetes and its vascular complications. Notes: Hazard ratios of univariate analysis are shown on the left side of the figure (Crude). Hazard ratios of a multivariate model with adjustment of sex, age and duration of diabetes (Model 1) are shown in the middle of the figure. Hazard ratios of a multivariate model with adjustment of Model 1 plus comorbid cancer, medications of diabetes, hypertension and dyslipidemia, habits of smoking and alcohol drinking, and frequency of physical activity (Model 2) are shown on the right side of the figure

Figure 1 shows the Kaplan‐Meier estimates for all‐cause mortality. The mortality in each vascular complication group was higher with statistical significance than that in the nonvascular complication group. Consistent with the mortality rates, patients with nephropathy had the highest mortality in the survival curves among the groups. Figure 2 shows crude and adjusted HRs of having vascular complications relative to not having them for mortality from various causes. In the two models with adjustment, the HRs of all‐cause (Model 1, *P* < .0001; Model 2, *P* < .0001), ischaemic heart disease (Model 1, *P* < .0001; Model 2, *P* < .0001), cerebrovascular disease (Model 1, *P* < .0001; Model 2, *P* < .0001) and infectious disease (Model 1, *P* < .0001; Model 2, *P* < .0001) mortality were significantly higher in the vascular complication groups than in the nonvascular complication group, and they were highest in the diabetic nephropathy group. While there were significantly high adjusted HRs for cancer mortality in the nephropathy and neuropathy groups relative to the nonvascular complication group, adjusted HRs in the macrovascular disease and retinopathy groups were not significantly high. As point estimates, adjusted HRs of all‐cause, ischaemic heart, cerebrovascular, and infectious diseases, and cancer mortality were highest in the diabetic nephropathy group.

For HRs of all‐cause mortality in Model 2, there were significant differences in the following pairs: macrovascular vs. nephropathy, macrovascular vs. no vascular complication, nephropathy vs. neuropathy, nephropathy vs. retinopathy, nephropathy vs. no vascular complication, neuropathy vs. no vascular complication and retinopathy vs. no vascular complication (all *P* < .05). For HRs of ischaemic heart disease mortality in Model 2, significant differences were detected in the following pairs: macrovascular vs. no vascular complication, nephropathy vs. retinopathy, nephropathy vs. no vascular complication, neuropathy vs. no vascular complication and retinopathy vs. no vascular complication (all *P* < .05). For HRs of cerebrovascular disease mortality in Model 2, significant differences were detected in the following pairs: macrovascular vs. no vascular complication, nephropathy vs. no vascular complication, neuropathy vs. no vascular complication and retinopathy vs. no vascular complication (all *P* < .05). For HRs of cancer mortality in Model 2, significant differences were detected in the following pairs: nephropathy vs. no vascular complication and neuropathy vs. retinopathy (all *P* < .05). For HRs of infectious disease mortality in Model 2, significant differences were detected in the following pairs: macrovascular vs. nephropathy, macrovascular vs. no vascular complication, nephropathy vs. neuropathy, nephropathy vs. retinopathy, nephropathy vs. no vascular complication, neuropathy vs. no vascular complication and retinopathy vs. no vascular complication (all *P* < .05).

## 4 DISCUSSION

In our study, Japanese patients with type 2 diabetes who were complicated by diabetic nephropathy showed point estimates of the highest mortality rates from all causes, cancer, infectious disease, renal failure and ischaemic heart disease. Unlike Western patients with diabetes who mostly died from ischaemic heart disease,^2^ Japanese patients mostly died from cancer. In Japanese patients, the second most common cause of death was infectious disease, and the third most common cause was ischaemic heart disease.

Western patients with diabetes and a history of macrovascular events have a high risk of mortality from recurrent macrovascular events.^13^ However, in our Japanese cohort, the point estimates of mortality rates (Table 2) and adjusted HRs (Figure 2) from ischaemic heart and cerebrovascular diseases in the nephropathy group were higher than those in the macrovascular disease group (Table 2). Previous studies have reported that patients with diabetic nephropathy^14^ and those with chronic kidney disease^15^ are at a higher risk of cardiovascular disease. Our Japanese data suggested that among various vascular complications, patients with diabetic nephropathy had the highest rate (Table 2) and four‐ to fivefold adjusted HRs of ischaemic heart disease mortality compared with those without vascular complications (Figure 2). Additionally, patients with diabetic nephropathy and macrovascular disease had the highest rate and three‐ to fourfold adjusted HRs of cerebrovascular disease mortality.

The prevalence of microvascular complications varies among countries and data sources. Previous studies investigated proportions of microvascular complications in Caucasian and Asian patients. In 2002, age‐ and sex‐adjusted analysis showed a larger prevalence of end‐stage renal disease in Blacks, Asians and Latinos than in Whites.^16^ Furthermore, a 2006 worldwide survey reported that the prevalence of Caucasian patients with nephropathy was 33%, while that of Asian patients was 43% (not including Japanese).^17^ A Japanese report included patients with type 2 diabetes with a mean age of 63 years and a mean duration of diabetes of 12 years, who were followed by diabetologists.^18^ This Japanese report showed that the prevalence of nephropathy of stage 1 or higher was 58%. However, our data from physicians with various specialties suggested that the prevalence of nephropathy was small (20.0%). This difference between studies may be partly explained by the fact that detecting diabetic nephropathy with microalbuminuria requires additional urine analysis as requested by physicians who care for patients with diabetes. In this previous Japanese report, diabetologists would have accurately diagnosed diabetic nephropathy at its onset in their patients. The prevalence of diabetic nephropathy of 20.0% in our data may have been underestimated in the setting of general hospitals. In another study, 26.4% of patients with type 2 diabetes in the UK were complicated by painful peripheral neuropathy.^19^ A US report described that at least 20% of adult patients have chronic sensorimotor distal symmetric polyneuropathy.^20^ In contrast, a Japanese study reported that 47.1% of patients with diabetes had diabetic neuropathy‐related symptoms, bilateral loss or a reduction in the Achilles tendon reflex, or bilateral reduction of sense vibration.^21^ Other Japanese studies showed that 36.7% of patients with type 2 diabetes had some type of diabetic neuropathy^22^ and 79.8% of patients with diabetes in the capital sphere had peripheral or autonomic neuropathy.^23^ The reason for the difference in the prevalence of diabetic neuropathy between previous Japanese studies^22^, ^23^ and our study (21.7%) may be that diagnosing diabetic neuropathy usually requires specific examinations by neurologists or diabetologists. By contrast, the prevalence of diabetic retinopathy is similar between a previous Japanese survey and our data. The prevalence of retinopathy was 40.3% in American patients with diabetes aged 40 years or older,^24^ while this prevalence was 22.9% in a report of Japanese patients with type 2 diabetes^22^ and 18.9% in our study. The reason for this similarity in Japanese data sets may be because diabetic retinopathy is relatively easily detected when older people consult ophthalmologists for problems with their eyes.

The life expectancy of Japanese patients with diabetes (71.4 years in men and 75.1 years in women^4^) is similar to that of American patients (71.3 years in men and 76.5 years in women^25^). However, the primary cause of death was cancer in our Japanese patients, as well as in Taiwanese patients with diabetes.^5^ This finding is in contrast to ischaemic heart disease as the primary cause of death in patients with diabetes in the US^26^ and in Italy.^27^ Indeed, in European and North American countries, although the incidence of acute myocardial infarction has been decreasing in the general population,^28^ the death rate of vascular disease in patients with diabetes is still markedly high (HR = 2.32, 95% confidence interval [95% CI]: 2.11‐2.56, vs. individuals without diabetes).^2^ Additionally, a previous study reported a large difference in coronary heart disease mortality rates between Japanese and American populations in the baseline years of 1992‐1993 (three‐ to fourfold death rate in American patients relative to Japanese patients).^29^ A hospital‐based death survey in Japan indicated that death from ischaemic heart disease in Japanese patients with diabetes greatly decreased from 10.2% from 1991‐2000 to 4.8% from 2001‐2010, with improved treatment of diabetes and its vascular complications.^4^ This survey also showed that death from ischaemic heart disease in the general population had slightly decreased from 7.3% to 6.5%. Therefore, the high mortality rate of cancer in our study could be explained by the decrease in mortality rate of ischaemic heart disease in Japanese patients with diabetes, a high mortality rate of cancer in the Japanese general population, and the extended life span of patients with diabetes who are vulnerable to cancer.

In our study, the highest sixfold mortality rate of ischaemic heart disease in patients with diabetic nephropathy compared with those with no vascular complications (Table 2) should be of concern to all healthcare professionals involved in diabetes. Surprisingly, patients with macrovascular disease did not have the highest HR, but had the second highest HR, for macrovascular mortality (Figure 2). Considering that the microvascular complication groups had relatively little overlap with the macrovascular complication group, the second highest all‐cause mortality rate of the macrovascular group should not have been greatly affected by patients with macro‐ and microvascular diseases at the same time. A study of Japanese patients with type 2 diabetes showed that a decreased estimated glomerular filtration rate was an independent risk factor of incident cardiovascular disease.^30^ This finding suggests that the severity of the estimated glomerular filtration rate may predict life expectancy of patients with diabetes. Consistent with the association between the estimated glomerular filtration rate and cardiovascular risk, in our cohort, patients with diabetic nephropathy had the highest mortality rates of ischaemic heart and cerebrovascular diseases, and all the major causes. Although vascular complications partly overlapped in our data, we found that the highest risk of death was in patients with diabetic nephropathy, rather than in those with a history of macrovascular complications. These results suggest that patients with diabetes need to prevent the development and progression of diabetic nephropathy to increase their life expectancy.

Our study showed little difference in cancer mortality rates among the vascular complication groups (Table 2 and Figure 2). Previous studies reported a 20% (95% CI: 15%–26%) extra risk of the incidence of cancer^31^ and a 25% (95% CI: 19%‐31%) extra risk of cancer mortality in patients with diabetes compared with individuals without diabetes.^2^ The reasons for these extra risks could be hyperinsulinemia^32^ or hyperglycaemia,^33^ and mortality of cancer increases in proportion to an elevation in fasting plasma glucose levels.^2^ Although we also aimed to determine the difference in cancer mortality rates among patients with diabetes with various vascular complications, we did not find a large difference in an extra risk of cancer mortality in those with and without vascular complications. This lack of difference could be attributed to similar HbA1c levels among the vascular complication groups, according to previous studies.^2^, ^34^

There are a few limitations of this study. First, the patients were limited to those who were followed up in medium to large hospitals. Because Japanese patients with mild diabetes are more likely to be followed up in clinics, the participating patients may have been biased towards those with more severe diabetes. This limitation could cause less generalizability of the current results; if we had included patients in clinics, the mortality rate would have been lower than that actually observed. In fact, the patients of our cohort were older than patients in other Western^2^ and Japanese^34^ studies. Additionally, the direction of this bias of the HRs relative to the nonvascular complication group is unknown when this limitation is present. However, when the outcomes were limited to mortality from various causes of death, which mostly occur in patients with moderate to severe diabetes (such as the present patients), the comparability of mortality rates and HRs among the vascular and nonvascular complication groups was relatively preserved. Second, while diabetic nephropathy in patients with diabetes can be detected by physicians who usually follow diabetes, diabetic retinopathy and neuropathy, which ophthalmologists and neurologist need to evaluate, are more likely to be underdiagnosed. This situation also implies that the nonvascular complication group might have included patients who actually had retinopathy or neuropathy and that the actual HRs of the retinopathy and neuropathy groups might have been higher than those measured in this study. In this case, the death risks of the retinopathy and neuropathy groups should be more strongly emphasized in clinical practice. Third, a subset of data of causes of death were missing. This lack of data reduced the sum of the mortality rates of major causes.

There are several aspects of our study that strengthened our results. First, we could calculate various cause‐specific mortality rates among patients with diabetes using vital statistics.^8^ Use of clinical records, interviews, and administrative data enabled a high follow‐up rate of 97.0% for the participants in the project. We consider that death of the patients was sufficiently recorded in this cohort. These results would be useful when clinicians prospectively quantify the prognostic mortality risks of their patients. Second, the number of participating patients with diabetes was large. This study may be unique in that the large sample size allowed estimation of cause‐specific or sex‐ and age‐specific mortality rates. Third, diagnoses of diabetes and its complications by medical doctors assured the internal validity and enabled comparisons among vascular complication groups.

In conclusion, data in Japanese hospitals show that among patients with diabetes, those with nephropathy have the highest mortality rates of all causes, cancer, and macrovascular and infectious diseases. The primary cause of death of these patients with diabetes with and without all vascular complications was cancer, and the cancer mortality rate was similar, regardless of whether vascular complications were present.

## CONFLICT OF INTEREST

The authors report no conflicts of interest in this work.

## AUTHOR CONTRIBUTIONS

Dr Nagai and Dr Hirata recruited the patients and helped follow‐up. Dr Yokomichi, Dr Mochizuki, Dr Hirata and Dr Kojima structured and wrote the paper. Dr Yokomichi and Dr Hirata analysed the data. Prof Yamagata oversaw the running of the study, enabling access to the patients’ clinical data and laboratory results. All co‐authors read and approved the final manuscript.

## ETHICAL APPROVAL AND INFORMED CONSENT

The ethics committee of the School of Medicine, University of Yamanashi approved this study (approval number: R1‐2040), in accordance with the ethical guidelines and regulations of the Declaration of Helsinki. All participants provided written informed consent to the project.

## Data Availability

The original data are available through a formal request to the BioBank Project.
